# Case report: Effectiveness of sirolimus in treating partial DiGeorge Syndrome with Autoimmune Lymphoproliferative Syndrome (ALPS)-like features

**DOI:** 10.3389/fped.2022.1014249

**Published:** 2023-01-18

**Authors:** Hao Gu, Wenjun Mou, Zhenping Chen, Xingjuan Xie, Jiafeng Yao, Rui Zhang, Runhui Wu, Jingang Gui

**Affiliations:** ^1^ Hematology Center, Beijing Key Laboratory of Pediatric Hematology Oncology, Beijing Children’s Hospital, Capital Medical University, National Center for Children’s Health, Beijing, China; ^2^ Laboratory of Tumor Immunology, Beijing Pediatric Research Institute, Beijing Children’s Hospital, Capital Medical University, National Center for Children’s Health, Beijing, China

**Keywords:** DiGeorge syndrome, ALPS-like, DNTs, mTOR, sirolimus monotherapy

## Abstract

**Background:**

DiGeorge Syndrome (DGS) is a rare disease associated with 22q11.2 chromosomal microdeletion, also known as a velocardiofacial syndrome, based on the frequent involvements of the palate, facial, and heart problems. Hematologic autoimmunity is rare in DGS but presents with a refractory course and poor prognosis. Herein, we report a case of partial DGS in a patient with refractory immune cytopenia and autoimmune lymphoproliferative syndrome (ALPS)-like manifestations.

**Case description:**

A 10-year-old boy with growth retardation presented initially with a ventricular septal defect at 7 months old, which had been repaired soon after. The patient suffered from thrombocytopenia and progressed into chronic refractory immune thrombocytopenia (ITP) at 30 months old. One year later, the patient developed multilineage cytopenias including thrombocytopenia, neutropenia, and anemia. First-line treatment of ITP, like high-dose dexamethasone and intravenous immunoglobulin, had little or short-term effect on controlling symptoms. Whole-exome sequencing revealed the presence of a *de novo* heterozygous 2.520 Mb deletion on chromosome 22q11.21. Moreover, decreased proportion of naive T cells and elevated double-negative T cells were found. The patient was given sirolimus therapy (1.5 mg/m^2^, actual blood concentration range: 4.0–5.2 ng/ml) without adding other immunosuppressive agents. The whole blood cell count was gradually restored after a month, and the disease severity was soothed with less frequency of infections and bleeding events. Decreased spleen size and restrained lymph node expansion were achieved after 3-month sirolimus monotherapy.

**Conclusions:**

This case is the first description on the efficacy of sirolimus monotherapy to treat refractory multilineage cytopenias of DGS presented with ALPS-like features.

## Introduction

DiGeorge Syndrome (DGS) is the most common chromosomal microdeletion disorder, caused by *de novo* nonhomologous meiotic recombination events and characterized by typical facial features. Based on their immunophenotype and degree of thymic hypoplasia, DGS is divided into a partial type and a complete type (1–3). Immunodeficiency, particularly impaired T-cell production, as a secondary consequence of diminished or lost thymic function affects up to 75% of pediatric DGS patients (4, 5). As one of the important features of immune dysregulation, increased TCRαβ^+^CD4^−^CD8^−^ double-negative T (DNT) cells and immune cytopenias are frequently seen in disorders with autoimmune lymphoproliferative syndrome (ALPS)-like phenotypes (6). Sirolimus is considered as an effective and safe therapeutic option for multilineage immune cytopenias with ALPS-like phenotypes (6). In light of the ALPS-like features with augmented DNTs in the DGS patient of our study, we envisaged that sirolimus, an inhibitor for the mammalian target of the rapamycin (mTOR) pathway, could possibly be used to treat the immune dysregulation in DGS, at least temporarily constraining the adverse consequences from immunodeficiency and the autoimmune manifestations (7). Here, we reported the efficacy and safety of sirolimus for treating a partial DGS patient with refractory autoimmune manifestations concomitant with increased DNTs.

## Case description

### Patient presentation

**Figure 1:**
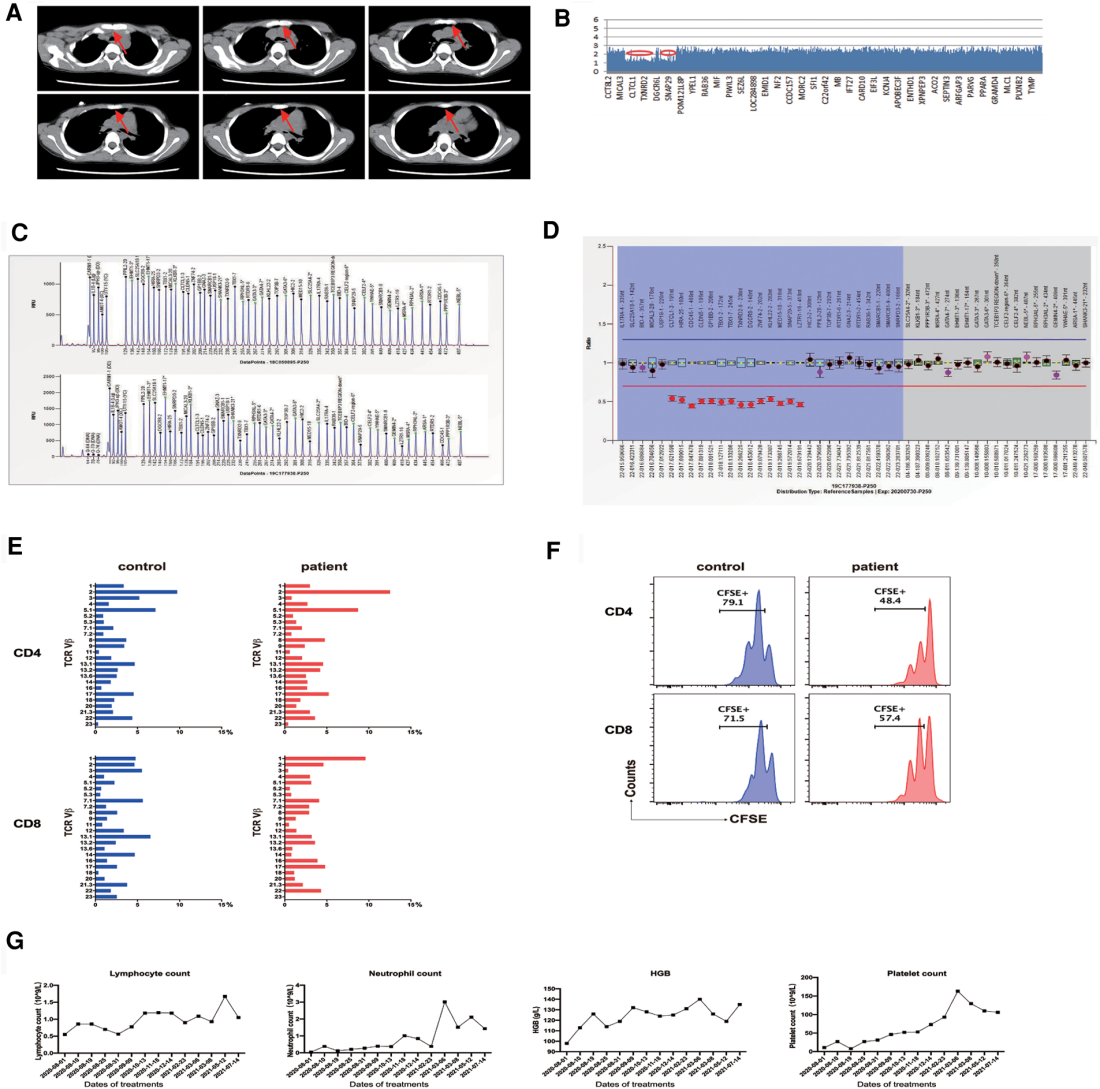
(**A**) Thymus volume is significantly reduced. (**B**) Whole-exome sequencing identifies a 2.520 Mb deletion on chromosome 22q11.21. (**C,D**) MLPA analysis reveals chromosome 22q11.21 deletion. (**E**) TCR Vβ repertoires analyzed by flow cytometry show a diverse and normal distribution of Vβ subfamilies. (**F**) Upon stimulation by anti-CD3/anti-CD28 for 4 days, CFSE-labeled CD4 and CD8 T cells from patient PBMCs show a retained proliferative response compared to a healthy control. (**G**) Changes in patient's lymphocyte, neutrophil, platelet, and hemoglobin (HGB) levels during sirolimus therapy. CFSE, carboxyfluorescein succinimidyl ester; MLPA, multiplex ligation-dependent probe amplification; PBMC, peripheral blood mononuclear cells; TCR, T-cell receptor.

The patient was a 10-year-old boy, born by a cesarean section at term to consanguinity-unrelated parents. The child was found to have a ventricular septal defect that was soon repaired by surgery at 7 months old. At age of 1.5 years old, thrombocytopenia was found (Plt 8 × 10^9^/L). The anti-glycoprotein (GP) IIb/IIIa test was positive, indicating severe bleeding (8). The bone marrow biopsy was normal, excluding the possible myeloid or lymphocyte-derived deformation. Based on the clinical features and cellular characteristics, immune thrombocytopenia (ITP) diagnosis was made. The symptoms were mitigated in response to glucocorticoid therapy (2 mg/kg bodyweight daily for 4 weeks). The patient unfortunately relapsed at 33 months of age and progressed to chronic refractory ITP. He suffered from splenomegaly at 6 years of age (5 cm below the left costal margin), after which massive lymphadenopathy was noticed. At 7 years of age, the patient developed neutropenia (0.15 × 10^9^/L) with low hemoglobin levels (99 g/L). The patient had a notable speech impediment and growth retardation. Computed tomography examination indicated a decreased thymus volume (Figure 1A). Serum IL-10 (8.28 pg/ml, reference range 1.2–4.55 pg/ml) was above the normal range, while parathyroid hormone was lower than normal (8.9 pg/ml, reference range 10.2–50.5 pg/ml). The ratio of lymphocyte subsets was abnormal (Table 1), with noticeably decreased naive T cells and elevated DNT cells (naive T cells, 9.5%, reference range 39.72%–65.59%; DNT cells, 4.4% of CD3^+^ T cells, reference range 0.82%–2.91%). The patient initially received first-line treatment for ITP (high-dose dexamethasone and intravenous immunoglobulin) with a poor response that did not last long. After monotherapy with sirolimus, the platelets and neutrophils recovered to a relatively normal level along with the disappearance of other clinical manifestations such as enlarged spleen and expanded lymph nodes.

**Table 1:** Lymphocyte subsets of the patient.

	Patient		Reference	
	%	Abs # (/µl)	%	Abs # (/µl)
Lymphocytes				
T cells				
DNT/CD3^+^	4.4	37	0.82–2.91	13–48
*γδ*T	3	26	8.10–20.76	124–388
CD3^+^	60.1	832	57.10–73.43	1325–2276
CD4^+^	24.3	337	24.00–38.72	531–1110
CD4 CM	78.5	265	24.24–52.73	165–475
CD4 naive	9.5	32	39.72–69.59	294–683
CD4 EM	11.8	40	3.40–11.17	24–87
CD4 TEMRA	0.2	1	0.10–1.29	0–9
Treg/CD4^+^	3.7	—	4.10–9.40	—
CD8^+^	31.1	431	21.01–33.94	480–1112
CD8 CM	26.3	113	13.21–37.89	92–287
CD8 naive	24	103	41.41–73.04	245–657
CD8 EM	36.7	158	1.52–15.39	9–130
CD8 TEMRA	13	56	2.01–21.65	12–164
B cells				
B cell	24.9	368	9.19–19.48	216–536
Naive B	19	70	51.84–77.61	123–362
Memory B	6.4	24	8.96–24.09	28–89
Plasmablast	0.4	1	0.7–5.67	3–21
Transitional	5	18	2.5–9.07	7–37

CM, central memory T cells; DNT, double-negative T cell; EM, effector memory T cells; TEMRA, terminally differentiated effector memory T cells; Treg, regulatory T cell.

### Genetic findings

Whole-exome sequencing (WES) revealed the presence of a *de novo* heterozygous 2.520 Mb deletion on 22q11.21 chromosome (18893867–21414817) (Figure 1B). To confirm the size of the missing area, multiplex ligation-dependent probe amplification (MLPA) analysis was performed with the standard MLPA kit (P250, MRC-Holland). The results revealed a 50% decrease relative to the reference bar height, indicating a heterozygous deletion (Figures 1C,D).

### Laboratory findings

The proportion and the absolute number of naive CD4 T cells were markedly decreased (proportion, 9.5%, reference 39.72-69.59%; cell number, 32 cells/μl, reference 294-683 cells/μl, Table 1). Determination of T-cell receptor (TCR) repertoires by flow cytometry revealed that the TCR repertoires of the patient were diverse and normally distributed in CD4 and CD8 T cells (Figure 1E). We then assessed the *in vitro* proliferative response of CD4 and CD8 T cells of this patient. Upon TCR ligation with anti-CD3/anti-CD28 antibodies, CD4 and CD8 T cells exhibited a retained potentiality of T cell division (Figure 1F). The patient was followed up for 1 year after sirolimus treatment, and the platelet count was recovered without any other hematologic abnormality being found. Consistent with the clinical improvements, his DNT cells decreased (from 4.4% to 3.2%) along with an elevation of regulatory T cells (Tregs, from 3.7% to 5.2%).

### Clinical course

Based on the medical history, clinical presentations (ventricular septal defect, thymus dysplasia, velopharyngeal hypoplasia, and delayed language development), genetic findings (chromosome 22q11.21 deletion), and laboratory data (decreased parathyroid hormone and reduction of naive T cells with a normal TCR Vβ repertoire), the patient was diagnosed with partial DGS.

The patient presented with elevated DNT cells (≥2.5% of CD3^+^ cells) and chronic (>6 months), nonmalignant, infection-free lymphadenopathy and splenomegaly, which are commonly seen in other ALPS-like disorders. Indeed, the patient had autoimmune cytopenias (hemolytic anemia, thrombocytopenia, and neutropenia) and hypoparathyroidism. Considering his autoimmune and lymphoproliferative symptoms, ALPS-like diseases such as activated PI3K-kinase delta syndrome (APDS) or CTLA-4 haploinsufficiency with autoimmune infiltration (CHAI) were considered (9). However, the WES results did not reveal defined variations associated with ALPS-like diseases. The WES results indicated that DGS, if not solely, would be the main contributor to ALPS-like phenotypes of the patient. In light of the clinical ALPS-like manifestations, sirolimus therapy was initiated (1.5 mg/m^2^, actual blood concentration range: 4.0–5.2 ng/ml) without the addition of other immunosuppressive agents. After 1 month of sirolimus treatment, the whole blood cell counts were gradually restored (Figure 1G), with a decrease in the severity and frequency of infections and bleeding episodes. Lymphadenopathy and splenomegaly were rapidly alleviated in this patient. The enlarged spleen shrunk from 5 cm below the left costal margin to 1 cm, highlighting encouraging results as early as 3 months from the start of sirolimus monotherapy.

## Discussion

Sirolimus has been increasingly recognized as an effective agent for ALPS patients and was reported to achieve a partial rescue of Tregs and suppression of DNT cells, which is consistent with our observations (10, 11). To some extent, applying sirolimus can achieve the sustainable recovery of immune cytopenias and splenomegaly, and the rebalance of abnormal immunophenotype. These data further suggest that sirolimus monotherapy is highly effective and may be beneficial for treating partial DGS with immune dysregulation associated with multiple cytopenias.

The clinical manifestations of DGS include hypoparathyroidism, conotruncal cardiac malformation, velopharyngeal insufficiency, facial dysmorphism, and intellectual disability. The immune dysregulation manifestations described in DGS include impaired antibody immune response resulting in poor response to vaccines and IgA deficiency (12–14). Autoimmune diseases such as juvenile rheumatoid arthritis, ITP, autoimmune hemolytic anemia, and Hashimoto thyroiditis are collectively common in DGS patients (14–17). The patient in the present study was shown to have an ALPS-like phenotype in many aspects, including decreased Tregs, increased IL-10 levels, and elevated DNT cells (6).

Recent findings showed that DNT cells are present in various chronic inflammatory diseases, including systemic lupus erythematosus (SLE), Sjögren's syndrome, psoriasis, axial spondylarthritis, and other rheumatic diseases as well (18). A clinical trial of sirolimus in patients with active SLE showed that mTOR blockade corrected proinflammatory DNT cell differentiation and activation (19). Furthermore, various studies have suggested that sirolimus is effective in multilineage cytopenias characterized by ALPS-like features. It has been suggested that increased DNT cells in ALPS-like diseases possibly come from autoreactive CD8 T cells via losing CD8 expression (19). Even though sirolimus monotherapy induced a reduction in DNT cells, it is not clear whether this is a secondary response from the holistically diminished autoimmune symptom or a direct cellular transformation in response to sirolimus.

In partial DGS, the primary thymic defect leading to impaired central tolerance was suggested because of autoimmune signs. Indeed, the absence of an appropriate central tolerance in partial DGS patients could lead to the escape of autoreactive thymocytes and the reduced absolute number and frequency of FoxP3^+^ thymocytes, consequently resulting in increased susceptibility to autoimmune manifestations (20). In recent works, increased FAS (APO-1/CD95) expression on lymphocytes and increased levels of FAS ligand (FASL) were found in patients with DGS (21). More efforts to understand the pathophysiology of partial DGS with ALPS-like symptom is expected.

## Conclusion

We reported a patient with partial DGS associated with clinically ALPS-like features, whose condition of refractory multilineage cytopenias was successfully treated with sirolimus monotherapy.

This case report emphasized that comprehensive laboratory diagnostic work is required for the accurate diagnosis of partial DGS. Otherwise, it could be easily misled by the immune-related cytopenias presented in other ALPS-like disorders. This case also showcases that sirolimus, as an effective drug for treating other ALPS-like disorders, is a good candidate for partial DGS patients, especially with autoimmune cytopenias and elevated DNTs.

## Data Availability

The raw data supporting the conclusions of this article will be made available by the authors without undue reservation.
